# Efficient transplacental IgG transfer in women infected with Zika virus during pregnancy

**DOI:** 10.1371/journal.pntd.0007648

**Published:** 2019-08-26

**Authors:** Tulika Singh, Cesar A. Lopez, Camila Giuberti, Maria L. Dennis, Hannah L. Itell, Holly J. Heimsath, Helen S. Webster, Hunter K. Roark, Paulo R. Merçon de Vargas, Allison Hall, Ralph G. Corey, Geeta K. Swamy, Reynaldo Dietze, Helen M. Lazear, Sallie R. Permar

**Affiliations:** 1 Duke Human Vaccine Institute, Duke University Medical Center, Durham, North Carolina, United States of America; 2 Department of Microbiology and Immunology, University of North Carolina at Chapel Hill, Chapel Hill, North Carolina, United States of America; 3 Núcleo de Doenças Infecciosas—Universidade Federal do Espίrito Santo, Vitoria, Espίrito Santo, Brazil; 4 Department of Pathology, Universidade Federal do Espίrito Santo, Vitoria, Espίrito Santo, Brazil; 5 Department of Pathology, Duke University Medical Center, Durham, North Carolina, United States of America; 6 Obstetrics and Gynecology, Duke School of Medicine, Durham, North Carolina, United States of America; 7 Global Health & Tropical Medicine—Instituto de Higiene e Medicina Tropical—Universidade Nova de Lisboa, Lisbon, Portugal; University of Pittsburgh, UNITED STATES

## Abstract

Zika virus (ZIKV) is a newly-identified infectious cause of congenital disease. Transplacental transfer of maternal IgG to the fetus plays an important role in preventing many neonatal infections. However, antibody transfer may also have negative consequences, such as mediating enhancement of flavivirus infections in early life, or trafficking of virus immune complexes to the fetal compartment. ZIKV infection produces placental pathology which could lead to impaired IgG transfer efficiency as occurs in other maternal infections, such as HIV-1 and malaria. In this study, we asked whether ZIKV infection during pregnancy impairs transplacental transfer of IgG. We enrolled pregnant women with fever or rash in a prospective cohort in Vitoria, Brazil during the recent ZIKV epidemic. ZIKV and dengue virus (DENV)-specific IgG, ZIKV and DENV neutralizing antibodies, and routine vaccine antigen-specific IgG were measured in maternal samples collected around delivery and 20 paired cord blood samples. We concluded that 8 of these mothers were infected with ZIKV during pregnancy and 12 were ZIKV-uninfected. The magnitude of flavivirus-specific IgG, neutralizing antibody, and vaccine-elicited IgG were highly correlated between maternal plasma and infant cord blood in both ZIKV-infected and -uninfected mother-infant pairs. Moreover, there was no difference in the magnitude of plasma flavivirus-specific IgG levels between mothers and infants regardless of ZIKV infection status. Our data suggests that maternal ZIKV infection during pregnancy does not impair the efficiency of placental transfer of flavivirus-specific, functional, and vaccine-elicited IgG. These findings have implications for the neonatal outomes of maternal ZIKV infection and optimal administration of antibody-based ZIKV vaccines and therapeutics.

## Author summary

In 2015, a Zika virus (ZIKV) epidemic emerged in Latin America, where dengue virus (DENV) already was endemic. The ZIKV epidemic revealed an array of birth defects and neurodevelopmental abnormalities in newborns associated with maternal infection. ZIKV may now be co-endemic in Latin America with DENV. Antibodies transferred from mother to the fetus in pregnancy can protect newborns from infections in early life, before they are eligible for vaccination. Conversely, flavivirus-specific IgG transfer could mediate enhancement of DENV infections in early life, or transfer ZIKV immune complexes into the fetal compartment. As a first step in evaluating these potential outcomes, it is important to understand whether ZIKV infection in pregnancy and its associated placental pathology impacts the magnitude or types of IgG subpopulations that are transferred across the placenta. To test this, we assessed paired maternal and cord blood collected at delivery from mothers who presented with rash and/or fever in pregnancy during the ZIKV epidemic in Vitoria, Brazil. Of these, we classified 8 as ZIKV-infected based on virus detection and/or neutralization serology, and 12 as ZIKV-uninfected. Comparing ZIKV-infected and uninfected groups, we detected no difference in transfer efficiency of IgG targeting ZIKV, DENV, or routine vaccine antigens. These findings indicate that the magnitude of IgG transferred across the placenta was not deficient at the time of birth in the setting of maternal ZIKV infection. Sustained transplacental IgG transfer with ZIKV infection during pregnancy indicates that ZIKV exposure in utero should not impact maternal antibody mediated protection during early life. However, concern remains over potential risk of severe primary DENV infection in ZIKV-exposed infants in endemic regions, or whether ZIKV could access the fetal compartment via antibody-mediated transport. This passive antibody transfer in pregnancy is an important consideration for flavivirus vaccine and therapeutic development efforts.

## Introduction

The emergence of Zika virus (ZIKV) in the Americas in 2015 revealed that ZIKV could be congenitally transmitted and cause fetal neurological damage [1–3]. Neurodevelopmental defects associated with congenital Zika syndrome (CZS) include microcephaly, arthrogryposis, motor and cognitive impairment, as well as vision and hearing loss [4]. ZIKV is the first example of a teratogenic vector-borne disease in humans. Initial estimates during the epidemic detected a 42% rate of fetal or neonatal abnormalities in symptomatic ZIKV-infected pregnant women [1], whereas subsequent epidemiologic studies with larger populations estimated a 7–14% rate of neurological defects in infants of pregnant women infected with ZIKV [5,6]. While the recent global epidemic has largely waned, the lack of preventative options for protection against ZIKV suggests ZIKV is likely to be a re-emerging and ongoing cause of congenital infections.

Transplacental transfer of IgG during pregnancy provides passive immunity to the fetus and is critical to protecting newborns against infections [7]. Maternal immunization during pregnancy can boost levels of protective IgG transferred to the fetus, providing a valuable tool for reducing neonatal morbidity. For example, tetanus immunization of pregnant women, or women of child-bearing age, resulted in a 94% reduction in neonatal tetanus mortality rates [8]. Moreover, maternal influenza vaccination and the magnitude of maternally derived antibodies are associated with protection of infants from influenza illness [9–11]. These benefits have led to the recommendation of providing diphtheria, tetanus, pertussis combined vaccines and influenza vaccines routinely during pregnancy [12,13]. Therefore, transplacental transfer of IgG is an important feature of maternal vaccination and natural immunity that may be leveraged for protection against neonatal pathogens.

Humoral immunity is thought to play an important role in protection against flavivirus infections [14–16]. ZIKV neutralizing antibodies likely provide durable protection against re-infection, therefore eliciting robust antibody responses is a key goal of ZIKV vaccine development [17]. Given the severe consequences of ZIKV disease in neonates, an ideal ZIKV vaccine would not only prevent infection in vaccine recipients but also protect fetuses from ZIKV congenital transmission. One way to protect fetuses could be transplacental transfer of ZIKV vaccine-elicited IgG. However, transplacental transfer of flavivirus-specific IgG also can lead to enhanced DENV disease during infancy, and may mediate transcytosis of ZIKV immune complexes [18–20]. Due to the key role of antibody transfer for newborn health, it is important to delineate the quantity and function of IgG transferred from mother to infant during pregnancy and to determine how transfer is altered by congenital pathogens.

The cross-reactive antibody responses between the antigenically similar DENV and ZIKV may lead to risks in early life for DENV disease enhancement in infants through transplacental transfer of flavivirus antibodies [19,21–24]. This risk is known to be mediated by antibodies generated from a prior DENV infection that can enhance DENV viremia and disease and ZIKV antibodies may have the potential to similarly enhance DENV infection [25–27]. Timing of past flavivirus infection also influences this risk as cross-neutralization of DENV and ZIKV is restricted to early convalescence, and antibody populations become more virus-specific over time [28,29]. While DENV-specific IgG are efficiently transferred in healthy pregnancies, waning maternal flavivirus-specific IgG levels throughout the first year of life leads to age-associated increased risk for severe DENV infection [19,30,31].

A second concern regarding placental IgG transfer is the potential of viral transcytosis from maternal to fetal compartment with immune complexes and subsequent enhanced fetal infection. Viruses such as human cytomegalovirus (HCMV) may co-opt this IgG transfer mechanism and traverse the placenta through the the neonatal Fc receptor (FcRn)[32,33]. Recent work suggests that ZIKV infection of human placental explants can be enhanced by DENV antibodies [18,34]. However, available epidemiogical data suggest that recent DENV infection provides modest protection against ZIKV[35,36], highlighting the need to better understand the impact of cross-reactive antibodies in flavivirus disease. Antibody-dependent transfer of ZIKV across the placenta, antibody-mediated enhancement of DENV disease in infants, and antibody-mediated protection of fetuses and newborns are all dependent on intact transplacental IgG transfer.

Maternal-fetal IgG transfer occurs at placental villus trees in contact with maternal blood [33]. In healthy pregnancies, IgG is transferred efficiently such that IgG concentrations in infant cord blood are often equivalent to or higher than the mother’s levels at delivery [7,37]. Many factors contribute to the efficient transplacental IgG transfer via FcRn, such as IgG subclass, antibody avidity, gestational stage, hypergammaglobulinemia [33,38,39], and maternal conditions or placental pathology.

Therefore, the premise for studying transplacental IgG transfer in the context of ZIKV infection in pregnancy is twofold. Firstly, maternal HIV-1 infection and placental damage due to malaria infection are two clinical settings associated with impaired IgG transfer [40–44]. Maternal ZIKV infection also results in placental damage, possibly due to viral infection of multiple placental cell types and inflammatory immunopathology [45–48]. Interestingly, maternal infection with DENV, a closely related flavivirus, leads to increased risk of maternal mortality, pregnancy complications, premature birth, and low infant birth weight, as well as placental damage [49–53]. Yet, DENV infection in pregnancy does not impair transplacental IgG transfer in normal birth weight infants [54]. ZIKV infection in pregnancy can result in prolonged viremia, suggesting a viral reservoir in an immune privileged site [55–58]. Secondly, maternal malaria and HIV-1 infection have been established to differentially impact transfer of IgG subpopulations specific to routine pediatric vaccines [40,59], which may be dependent on distinct Fc characteristics of each IgG population [60]. This phenomenon impacts antibody half-life in infant circulation and protection in early life. While prior studies show efficient transfer of recently boosted flavivirus antibodies after the ZIKV epidemic [61], we further examined whether pre-existing IgG subpopulations relevant to newborn health are efficiently transferred following maternal ZIKV infection.

To investigate whether ZIKV infection during pregnancy impairs transplacental transfer of IgG specific to flaviviruses and common vaccine antigens, we enrolled a prospective cohort of 26 pregnant women from Vitória, Brazil, who presented with fever and rash symptoms consistent with ZIKV infection during the recent Brazilian ZIKV epidemic. Of these women, 20 paired maternal plasma and infant cord blood samples were available from delivery and used to define the efficiency of transplacental IgG transfer. Evaluating the magnitude and subpopulations of IgG transferred to newborns who are exposed to ZIKV in utero is critical to understanding the extent of vaccine protection or risk of severe flavivirus infections in early life, and the development of antibody-based therapeutics.

## Methods

### Study population and design

This study enrolled 26 pregnant women living in Southeast Brazil, from which only 20 delivery samples were collected. All enrollees presented with fever and/or rash during the ZIKV epidemic to investigate maternal and infant immunity to ZIKV infection during pregnancy. Two groups of mother-infant pairs are included in this observational study: one group with maternal ZIKV infection during pregnancy, and the other group without ZIKV infection during pregnancy. Therefore, mothers with fever or rash during pregnancy but without ZIKV infection served as a comparator group for those with ZIKV infection and symptomology.

Participants in this study were enrolled from July 2016 to October 2017 in the city of Vitória, which is the capital of the State of Espírito Santo. There are 4 million inhabitants and 50,000 births per year in Espírito Santo with the majority living in the metropolitan region of Vitória [62,63]. This region has had endemic DENV circulation for the past two decades [64] so it was expected that many participants would have been exposed to DENV previously and be seropositive for DENV. The first clinically suspected cases of ZIKV infection in Brazil were described in May 2015, and six months later (November 2015) the first autochthonous ZIKV case was confirmed in Espírito Santo [65–67]. In the months preceding our enrollment, there was a ZIKV incidence of 3,100 cases per 100,000 inhabitants, and a DENV incidence of 901 cases per 100,000 inhabitants in Espírito Santo [64]. In this timeframe, 77 CZS cases were reported to the State Health Department, including cases of microcephaly, defects of the central nervous system suggestive of congenital infection, or stillbirths [64]. Since this region reflected key features of flavivirus co-endemic settings and had ongoing ZIKV transmission, it was considered representative of regions with a burden of ZIKV disease and appropriate for study of maternal and infant ZIKV immunity.

### Recruitment

The enrollment field site is based in the city of Vitória at the the Núcleo de Doenças Infecciosas (NDI), at the Universidade Federal do Espírito Santo. During our study, suspected ZIKV infection was considered a reportable condition to the State Health Department for all patients seen at public or private clinics within the state. Within a week of a case reported by a physician to the State Health Department, a staff member reported notifications of pregnant suspected ZIKV cases within the State to NDI. Thus the recruitment strategy relied on passive surveillance systems, and no active recruitment was conducted in the community. Upon referral, staff at the NDI contacted pregnant suspected ZIKV cases within the Vitória metropolitan area by phone regarding interest in participating in this study. If interested, pregnant suspected ZIKV cases were invited to the NDI for written informed consent and first recorded visit in our study at the time of enrollment.

### Enrollment and follow-up

At the initial visit for study enrollment, three inclusion criteria were confirmed: 1) pregnant women with rash or fever; 2) patient was a minimum of 18 years of age; 3) willingness to participate in study through provision of written informed consent. No exclusion criteria were defined. During the enrollment visit, a clinical history and physical evaluation were performed by a licensed physician, and blood and urine were collected. The following demographic information was collected at enrollement: age, municipality, date of birth, last menstrual date, recall of prior DENV disease, family members or neighbors with symptoms of ZIKV infection, use of insect repellant, prior vaccination for yellow fever virus, sexual activity in the 10 days before symptoms of ZIKV infection, symptoms of ZIKV infection in sexual partners, partner’s use of insect repellent, and use of drugs, tobacco, or alcohol during pregnancy. Any clinical records and ultrasounds during the pregnancy before symptoms of ZIKV infection also were collected. All participants were referred for additional prenatal clinical care consultations and ultrasounds. Transportation to the NDI research site for every visit, as well as all recommended consultations with obstetrician-gynecologists and ultrasounds were funded by the study. For each participant, gestational age at the time of symptoms and delivery was calculated based on the last menstrual period date and confirmed by ultrasound (performed at 9–22 weeks).

After the enrollment visit, all participants were followed up weekly for up to four weeks, and monthly visits thereafter until delivery. Though followup of the mothers and infants in this study is ongoing, the present report only includes samples through delivery. At every visit, a standardized questionnaire was administered in the form of a semi-structured interview by a trained research staff member at NDI. Through this questionnaire we collected information on the presence and duration of symptoms related to ZIKV infection.

At the time of delivery, maternal blood and urine, infant cord blood, and placenta were collected. Newborn head circumference was measured by a nurse prior to hospital discharge, and reported to study staff. Head circumferences were converted to z score for the corresponding gestational age using the Newborn Cross-Sectional Study of the INTERGROWTH-21st Project standards. Microcephaly was defined per WHO and INTERGROWTH-21^st^ guidelines as a z score lower than -1.88, which is the 3rd percentile of newborns at each gestational age [68,69].

### Sample collection

Blood samples were collected into heparin or EDTA tubes, stored at room temperature up to six hours, and centrifuged at 1300 x G for 10 minutes to obtain plasma. Infant umbilical cord blood was collected by clamping the cord, cutting it, and draining blood into sterile collection tubes. Urine samples were collected mid-stream in a sterile screw-top container and stored at -80°C. Plasma samples were stored at -80°C, then shipped to Duke University on dry ice.

### Ethics statement

This prospective cohort study was approved by the Institutional Review Board of Hospital Cassiano Antonio Moraes, Brazilian National Research Ethics Committee (CEP/CONEP Registration number: 52841716.0.0000.5071), and Duke University Medical Center Institutional Review Board (Pro00100218). Women meeting enrollment criteria who provided written informed consent were included.

### RT-PCR assay for detection of ZIKV

Viral RNA was extracted from 140μL of plasma and urine using QIAmp Viral RNA Mini Kit (Qiagen). Previously described RT-PCR primers and probes specific for ZIKV were used: ZIKV1086, ZIKV 1162c, and ZIKV1107-FAM [70]. For this one-step RT-PCR reaction, 5μL of RNA was combined with 500nM primers, 250nM probe and nucleotides in a total volume of 20μL, including SuperScriptIII RT and Platinum Taq DNA polymerase Mix (Invitrogen). The negative controls were serum from a 30-year old asymptomatic subject in Vitoria collected in 2016, and PCR grade water (no template control). The positive control was supernatant from ZIKV-infected Vero cells. Samples and controls were tested in duplicate, and ZIKV positivity was indicated by detection of amplification at <38 cycles in both duplicate wells on the Applied Biosystems 7500 Fast platform.

### ZIKV IgM antibody capture enzyme-linked immunosorbent assay (MAC-ELISA)

The CDC MAC-ELISA was adapted and used to detect IgM specific for ZIKV in maternal and cord blood plasma [71]. Briefly, 96-well high-binding ELISA plates were coated with 20 μg/ml of mouse anti-human IgM (Sigma #I0759) overnight at 4°C. Plates were blocked for 30 minutes at room temperature with 5% milk in 0.5% TBST, and then samples were added at a 1:40 dilution in quadruplicate for 1 hour at 37°C. Antigen (ZIKV H/PF/2013 grown in C6/36 cells), or C6/36 conditioned media as a negative control, was added at a 1:40 dilution overnight at 4°C. Then, an HRP-conjugated pan-flavivirus antibody (6B6C-1) was added for 1 hour at 37°C, followed by TMB substrate. Plates were incubated for 20 minutes, upon which 1N H_2_SO_4_ was added to stop the reaction. A positive result required that the absorbance for a particular plasma was greater than 3-fold higher than the absorbance for that same plasma on C6/36 conditioned media. Samples run on each plate also include a confirmed ZIKV IgM positive and negative sample.

### Cell culture and virus stocks

Vero-81 cells were grown in Dulbecco’s Modified Eagle Media (Gibco 11965092) supplemented with 5% heat-inactivated fetal bovine serum (Cellgro, Cat#35-016-CV) and L-alanyl-L-glutamine (Thermofisher, GlutaMAX Cat#35050079). Viruses used for the focus reduction neutralization test were DENV1 (WestPac74), DENV2 (S-16803), DENV3 (CH54389), DENV4 (TVP-360), obtained from Dr. Aravinda de Silva, University of North Carolina at Chapel Hill, and ZIKV (H/PF/2013), obtained from the United States Centers for Disease Control and Prevention (Division of Vector-borne Diseases, Fort Collins, CO). For the detection of virion binding antibodies, the following viruses from BEI were used: ZIKV (PRVABC59), DENV1 (Hawaii), DENV2 (New Guinea C), DENV3 (Philippines), and DENV4 (H241). Virus stocks were grown in Vero-81 cells supplemented with 2% heat-inactivated fetal bovine serum and 10mM HEPES (Corning, Cat#25-060-CI).

### Placental sampling and examination

Placenta samples were available from 11 ZIKV-infected and 8 ZIKV-uninfected subjects out of 26 mothers total in the cohort. Fragments were collected from the whole placenta up to 24 hours after delivery. Three sets of full thickness samples of placental parenchyma were obtained in every case and histology performed as previously described [72]. For the histological analysis, sections were fixed in 4% formaldehyde phosphate buffered solution, paraffin embedded, and 5μm sections were stained with hematoxylin and eosin. Histological sections were examined specifically for villous lesions by a pathologist. Villitis was diagnosed if inflammatory exudate was present in the trophoblast or in the villous stroma and was categorized by Knox & Fox and Redline criteria [73,74]. Placentas were assessed as low-grade villitis if less than 10 villi were involved per focus, and high-grade if more than 10 villi were involved per focus [73].

### Focus reduction neutralization test

We used previously described methods for FRNT-50 in a 96 well plate [29]. Briefly, serial 5-fold dilutions of heat-inactivated plasma were added to 50–80 focus forming units of either DENV or ZIKV and incubated for 1 hour at 37°C, then transferred to a confluent plate of Vero-81 cells and incubated for 1 hour at 37°C. Then an overlay of 1% methylcellulose was added. Cells were fixed with 2% paraformaldehyde and stained with 1 μg/mL of E60 mouse monoclonal antibody targeting the conserved flavivirus fusion loop [75], then detected with an anti-mouse IgG horseradish peroxidase conjugate and True Blue substrate (KPL). FRNT-50 values were calculated with the sigmoidal dose-response (variable slope) curve in Prism 7 (GraphPad), constraining values between 0 and 100% relative infection. A valid FRNT-50 curve required an R^2^ >0.75, hill slope absolute value >0.5, and had to reach at least 50% relative infection within the range of the plasma dilutions in the assay.

### Detection of virion binding IgG

To measure IgG binding responses against whole flavivirus virions, high-binding 96-well ELISA plates (Greiner) were coated with 30 ng/well of 4G2 antibody (clone D1-4G2-4-15) in carbonate buffer, pH 9.6 overnight at 4°C. Plates were blocked in Tris-buffered saline containing 0.05% Tween-20 and 5% normal goat serum for 1 hour at 37°C, followed by an incubation with either ZIKV, DENV1, DENV2, DENV3 or DENV4 for 1 hour at 37°C. Plasma was tested at a 1:25 starting dilution in 8 serial 3-fold, 5-fold, or 10-fold dilutions, incubating for 1 hour at 37°C. Horseradish peroxidase-conjugated goat anti-human IgG antibody (Jackson ImmunoResearch Laboratories, Inc; 109-035-008) was used at a 1: 5,000 dilution, followed by the addition of SureBlue reserve TMB substrate followed by stop solution (KPL). Optical densities (OD) were detected at 450 nm (Perkin Elmer, Victor). ED_50_ values were calculated with the sigmoidal dose-response (variable slope) curve in Prism 7 (GraphPad), which uses a least squares fit. An ED_50_ value was considered valid if the OD at plasma dilution 1:25 was two (2SD) or three (3SD) standard deviations above the mean OD observed for 11 plasma samples from healthy U.S. subjects (2SD OD cut-offs: DENV-1 = 0.406, DENV-2 = 0.648, DENV3 = 0.906, and DENV-4 = 0.885; 3SD OD cut-off: ZIKV = 0.596). Software generated ED_50_ values from curves with an OD at 1:25 plasma dilution below this cut-off were considered non-binding and plotted at the limit of detection.

### Determination of transplacental transfer of IgG against routine pediatric vaccines

IgG binding to antigens from pediatric vaccines that are used routinely in Brazil was tested using a customized binding antibody multiplex assay on the Luminex platform, as previously described [76]. Pediatric vaccine antigens used for screening included: hepatitis B virus surface antigen (antigenic combination: adw), rubella virus capsid (AbCam), *Bordetella pertussis* toxin and *Corynebacterium diphtheriae* toxin (Sigma-Aldrich), *Haemophilus influenzae* type B oligosaccharide-conjugated to human serum albumin (HbO-HA), and tetanus toxoid (Reagent Proteins). Antibody binding was detected with mouse anti-human IgG-PE (Southern BioTech) and the fluorescent output was measured on a Bio-Plex 200 system (Bio-Rad Laboratories). Antibody concentrations in μg or International Units per mL were interpolated from corresponding sigmoidal curves of serially diluted WHO international reference sera (National Institute of Biological Standards and Control, Potters Bar, UK; NIBSC code numbers: 07/164, 09/222, 06/140, TE-3, 10/262, RUBI-1-94). The efficiency of transplacental IgG transfer was calculated for each mother-infant pair by dividing the concentration of infant pediatric vaccine-specific IgG by the concentration of maternal vaccine-elicited IgG.

### Screening for neonatal TORCH pathogens

Data on Toxoplasma, rubella, and syphilis serological status was extracted from the mother’s prenatal visit clinical records. All tests were performed by State Health Department or clinical laboratories using commericially available kits approved by the Brazilian Health Regulatory Agency (ANVISA), as per the manufacturer’s instructions. Chemiluminescent microparticle immunoassay kits were utilized for detection of Toxoplasma IgM and IgG, as well as rubella virus IgG. Syphilis serostatus was assessed using a Venereal Disease Research Laboratory test, which is a nontreponemal test. Congenital HCMV infection was evaluated in our research laboratory using quantitative PCR of infant cord blood. To pellet HCMV from plasma, 200 μL of infant cord blood was transferred to a high g-force micro-centrifuge tube and spun in an S45A fixed angle rotor at 30,000 rpm, 4°C, for 3 hours in a Sorvall Discovery M120 Ultracentrifuge. Then the supernatant was removed and the pellet re-suspended in 200 μL of 1x PBS. DNA was extracted using the Roche High Pure Viral Nucleic Acid Kit according to the manufacturer’s protocol. To quantify and detect HCMV DNA, extracted DNA from each sample was amplified in six replicates. For this reaction, 5 μL of DNA was added to 15 μL SYBR Select Master Mix with (ThermoFisher Scientific), 5 μL of water, and 300 nM primers designed to amplify the immediate-early 1 (IE1) gene of HCMV (Integrated DNA Technologies). IE1 Forward Primer (20 bp): CAA GCG GCC TCT GAT AAC CA. IE1 Reverse Primer (24 bp): ACT AGG AGA GCA GAC TCT CAG AGG. For the negative control, PCR grade water was used as a substitute for extracted DNA in the reaction with in four replicate wells. A 10-fold, 7 series dilution of plasmid with the amplification region was serially diluted starting at 1x10^8^ copies/mL to generate a standard curve for quantitation of HCMV DNA in each sample. The lowest dilution on the standard that could be reliably amplified across replicates was considered as the threshold for positivity (250 viral DNA copies/mL).

### Definition of ZIKV infection

**Fig 1:**
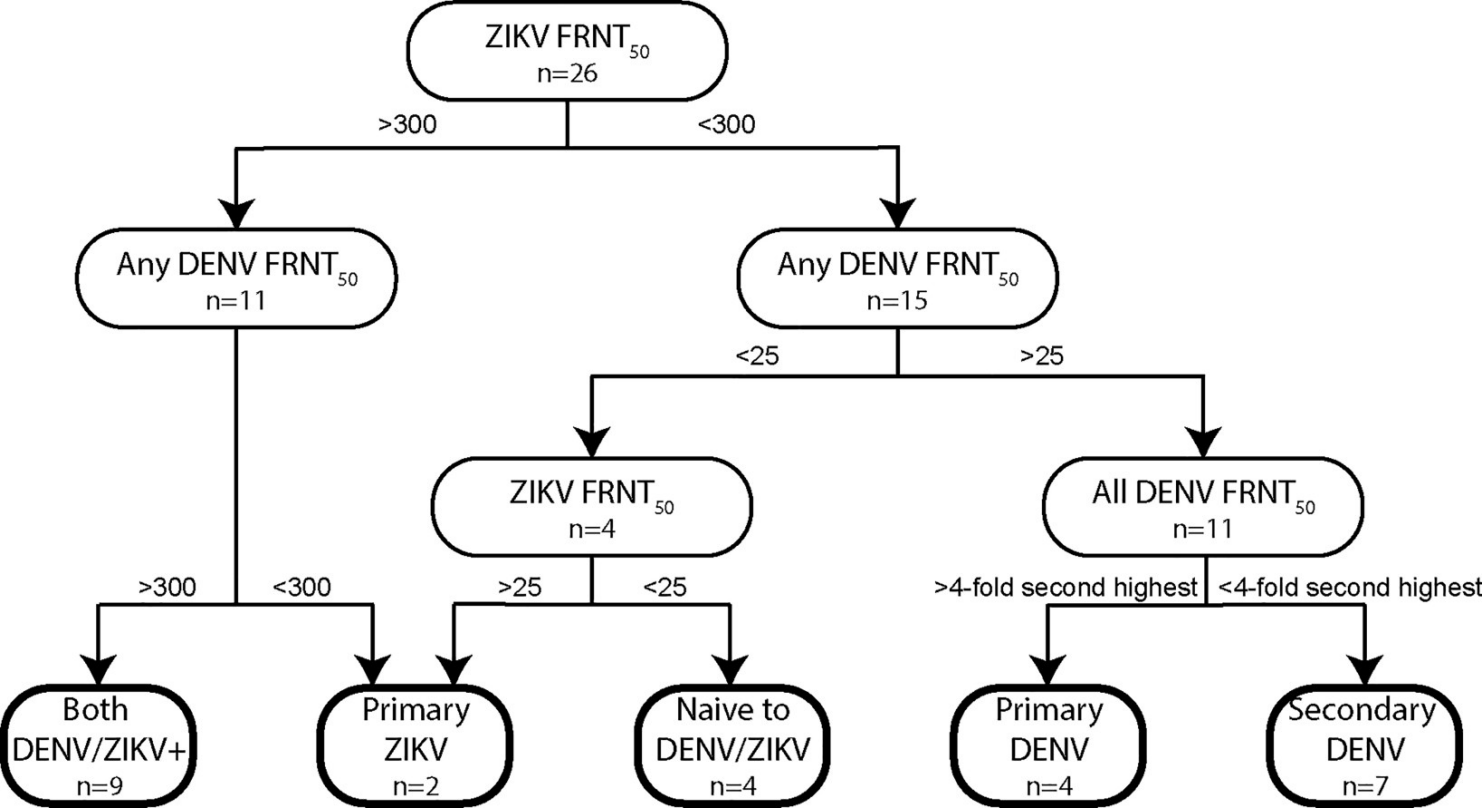
Algorithm used to categorize flavivirus exposure history according to ZIKV and DENV focus-reduction neutralization-50 titers (FRNT-50). Maternal and cord blood plasma were tested by FRNT50 against 5 viruses (ZIKV and 4 DENV serotypes) and FRNT-50 titers used to infer flavivirus exposure history. All samples were anti-flavivirus IgM negative, reducing the likelihood of cross-reactivity resulting from recent infections.

As ZIKV viremia is transient, RT-PCR does not reliably detect ZIKV infection beyond 10–14 days from exposure [77]. Therefore, we combined a RT-PCR diagnostic with serological approaches based on delivery maternal plasma FRNT-50 titer (FRNT-50) against ZIKV and DENV (types 1–4). “Primary ZIKV” infection (no prior DENV or ZIKV infection) was defined as either i) a high ZIKV FRNT-50 (>300) and a low DENV1-4 FRNT-50 (<300), or ii) a low ZIKV FRNT-50 titer that is still >25 and at least one DENV FRNT-50 >25, suggesting only a weak transient cross-neutralizing response between ZIKV and DENV. A history of both ZIKV and DENV (“DENV+ZIKV”) was defined as high ZIKV FRNT-50 (>300), and at least one DENV FRNT-50>300. DENV immunity only (no ZIKV immunity) was classified as low ZIKV FRNT-50 (<300), but DENV FRNT-50 >25 (Fig 1). Thus, we defined ZIKV infection as “primary” or “secondary” ZIKV based on serological evidence of prior DENV exposure, whereas the ZIKV-uninfected group may include subjects naïve to both ZIKV and DENV or those exposed to only DENV.

Since infection with one DENV serotype results in neutralizing activity against that same serotype [78], and a subsequent infection with a different serotype results in broad DENV cross-neutralizing activity, we designed criteria to differentiate primary and secondary DENV infections based on whether the second-highest DENV FRNT-50 was within four-fold of the highest DENV FRNT-50. To further account for serological cross-reactivity from recently infected subjects in assessing ZIKV infection status, we confirmed DENV-negative status by RT-PCR where acute samples were available. Sera with FRNT-50 values below the limit of detection for all five viruses were classified as ZIKV and DENV naïve. This definition was based on the assumption that a dominant ZIKV neutralization response at delivery was attributable to the recent symptomatic illness during pregnancy and not a prior ZIKV infection, given the recency of ZIKV introduction to the region during the period of enrollment. RT-PCR results from a plasma sample collected <7 days after symptom onset that were discordant with the serological assessment were repeated.

### Statistical analysis and power

Statistical analysis was performed using SAS (version 9.4) and Prism software (GraphPad; version 7). Serological responses are presented as a magnitude of flavivirus binding IgG (ED50), neutralizing (FRNT-50), and vaccine antigen binding IgG (μg/mL or IU/mL). These measures were assessed for each of the 26 maternal and 20 infant delivery samples, for each antigen tested (S1 Fig). The percent IgG transferred from mother to infant describes the transplacental transfer efficiency, and is calculated as the ratio of the magnitude of infant cord blood IgG binding level (measured as ED50 or μg/ml) to the maternal IgG binding level multiplied by 100. Note that this percent transfer ratio is specific to each antigen tested. Data are presented as dot plots of percent transfer for each mother infant pair in the ZIKV-infected group as compared to the ZIKV-uninfected group. Scatter plots are used to display the relationship and distribution of the maternal IgG level as compared to the infant IgG level, by antigen.

With a sample size of 26 mothers and 20 infant samples, our study is powered to reject the null hypothesis (no correlation between maternal and infant antibody responses), at an alpha of 0.05 with a power of 0.89 for neutralizing titer correlations, and 0.99 for correlations of IgG binding to flaviviruses or vaccine antigens. Therefore, this study is adequately powered to detect associations between maternal and infant antibody measures. For Wilcoxon Rank tests comparing IgG transfer efficiency between ZIKV-infected and uninfected mothers, this study is powered to assess significant differences between ZIKV-infected and uninfected groups in flavivirus IgG binding at an alpha of 0.05 (power = 0.93), but not for vaccine antigen IgG (power = 0.15) and neutralizing IgG (power = 0.48). This is due to differences in the extent of variability in measures by assay type.

Due to the small size of this cohort, a Gaussian distribution could not be inferred and therefore non-parametric statistical tests were applied. To compare IgG binding between ZIKV-infected and -uninfected groups, the Wilcoxon Signed and Exact Wilcoxon Rank Sum tests were applied. For correction of multiple comparisons, the Bonferroni correction was applied. Data were not stratified beyond the ZIKV infection status exposure group. The Kendall Tau test was used to evaluate correlations between maternal and infant responses with the alpha level of significance set to 0.05.

## Results

### Cohort characteristics

Pregnant women aged 18 to 39 years were enrolled based on symptoms suggestive of ZIKV infection, such as rash, arthralgia, and fever (Table 1). Nearly all enrolled participants (24/26) were from the Vitoria metropolitan area. One subject (B1_0037) exhibited prolonged viremia, which was detected by RT-PCR up to 42 days post symptoms. Mothers were tested for common congenital “TORCH” pathogens where samples were available (S1 Table). These data indicate no recent *Toxoplasma* infections (no maternal IgM positive sera), high IgG seropositivity to rubella virus, and no evidence for maternal syphillis infection. Testing of infant cord blood for HCMV DNA found one case of congential HCMV transmission in the ZIKV-uninfected group.

**Table 1:** Symptomatology of patient cohort at the time of enrollment based on ZIKV detection by RT-PCR.

	ZIKV PCR+ (n = 9)	ZIKV PCR- (n = 13)	ZIKV PCR ND (n = 4)	Total (n = 26)
	9/26	13/26	4/26	26/26
**Proportion of mothers symptomatic in each gestational trimester**				
First	3/9	3/13	2/4	8/26
Second	4/9	7/13	2/4	13/26
Third	2/9	3/13	0/4	5/26
**Proportion with symptoms**				
Rash	8/9	13/13	3/4	24/26
Arthralgia	5/9	4/13	3/4	12/26
Fever	3/9	5/13	3/4	11/26
Conjunctivitis	4/9	4/13	3/4	11/26
Myalgia	5/9	4/13	1/4	10/26
Headache	4/9	7/13	3/4	14/26
Retro-orbital pain	2/9	4/13	2/4	8/26
Lymphadenopathy	1/9	1/13	0/4	2/26

ZIKV = Zika virus, RT-PCR = reverse-transcription polymerase chain reaction, ND = not done.

### Serologic profile of flavivirus neutralization

ZIKV testing by RT-PCR was performed in plasma and urine, collected between 2 and 15 days post symptom onset in 22 out of 26 women (Table 2). According to plasma neutralization titers, most women were DENV seropositive, regardless of ZIKV infection status. The remaining four women were referred for enrollment only after the resolution of symptoms, at 36 to 217 days since symptoms, and thus their negative ZIKV RT-PCR result was inconclusive. All women with acute samples available were negative for DENV by RT-PCR at enrollment, and one (B1_0035) was positive for CHIKV by RT-PCR (S2 Table).

**Table 2:** ZIKV and DENV serotype specific humoral immune profile. Serologic classification of maternal flavivirus infection history was determined by focus reduction neutralization titer 50% (FRNT-50) against ZIKV and DENV in plasma taken at delivery.

Sample ID	Classification	Days since symptoms	FRNT_50_					ZIKV RT-PCR
			ZIKV	DENV1	DENV2	DENV3	DENV4	
B1_0015	Naïve	NA	<25	<25	<25	<25	<25	-
B1_0019	Naïve	75	<25	<25	<25	<25	<25	-
B1_0021	Naïve	213	<25	<25	<25	<25	<25	-
B1_0039	Naïve	35	<25	<25	<25	<25	<25	ND
B1_0008	Primary ZIKV	184	3918	126	209	251	106	+
B1_0030	Primary ZIKV	77	1399	<25	<25	<25	<25	+
B1_0001	DENV+ZIKV	193	10858	898	597	1270	491	+
B1_0002	DENV+ZIKV	173	14959	1524	666	5571	502	+
B1_0004	DENV+ZIKV	164	2533	1348	1818	3047	537	+
B1_0005	DENV+ZIKV	217	5213	1379	4218	2270	359	+
B1_0007	DENV+ZIKV	208	5503	1371	2511	822	353	ND
B1_0014	DENV+ZIKV	210	3095	354	1625	930	388	-
B1_0031^a^	DENV+ZIKV	94	1610	2723	2492	10521	1510	+
B1_0027	DENV+ZIKV	91	654	1079	1711	3730	513	-
B1_0037	DENV+ZIKV	117	11764	2141	8019	22873	4029	+
B1_0009^b^	Primary DENV2	240	<25	205	1887	238	240	+
B1_0035	Primary DENV2	114	107	68	1201	106	68	-
B1_0011	Primary DENV3	39	<25	374	640	3172	362	-
B1_0006	Primary DENV3	211	<25	89	308	4735	82	ND
B1_0003	Secondary DENV	217	<25	797	122	304	72	-
B1_0016	Secondary DENV	172	<25	232	3222	2051	74	ND
B1_0023	Secondary DENV	92	<25	4417	1693	380	<25	-
B1_0024	Secondary DENV	146	<25	1395	1362	505	299	ND
B1_0026	Secondary DENV	46	<25	2848	1876	635	292	ND
B1_0033	Secondary DENV	91	220	3123	1996	843	197	-
B1_0034	Secondary DENV	111	<25	193	568	939	76	-

ZIKV RT-PCR performed on plasma collected at enrollment (median 146 days since symptoms).

ND = Not Done

^a^ FRNT-50 based on maternal plasma 3 months after delivery

^b^ Likely false positive ZIKV RT-PCR result

Because ZIKV viremia typically is detected only in the acute phase of infection (≤14 days after exposure), and the possibility of a false positive RT-PCR ZIKV test, we used serology to classify maternal ZIKV exposure as well as prior DENV infection history. Since detection of ZIKV-binding antibodies by ELISA does not distinguish ZIKV exposure from other flaviviruses, and this region has high DENV seroprevalence, we determined the FRNT-50 of all maternal plasma samples collected at delivery, which ranged from 39 to 217 days following onset of ZIKV symptoms. Although DENV and ZIKV antibodies cross-react in binding assays (e.g. ELISA), we and others have shown that there is minimal cross-neutralizing activity in convalescent sera [23,29]. By these definitions, 11 out of 26 women had serological evidence of ZIKV infection, only 2 of which were DENV naïve, indicating a primary ZIKV infection (Table 2). Two out of 26 women were naïve for both ZIKV and DENV, and the rest had serological evidence of DENV infection with no ZIKV infection. Though one mother classified as ZIKV naïve (Primary DENV) by serology (B1_0009) had a positive RT-PCR result at initial presentation, subsequent RT-PCR testing of stored plasma was negative, suggesting that the initial result was a false positive. Of note, two patients (B1_0002 and B1_0037) were ZIKV IgM positive at delivery.

### Infant outcomes

At birth, all infants born to ZIKV negative mothers were assessed to be healthy. Of the 11 infants born to mothers with serological evidence of ZIKV infection, one infant (born to B1_0001) presented with microcephaly at birth, with a head circumference below 3^rd^ percentile based on WHO International Standards, and neurologic abnormalities such as cortical-subcortical calcifications, dysgenesis of the corpus callosum, pachygyria, and colpocephaly upon transfontanellar ultrasound and CT scan [79]. Delivery cord blood sample was not available for this infant. Neurodevelopmental assessments of the infants from this cohort are ongoing.

### Placental histology

**Fig 2:**
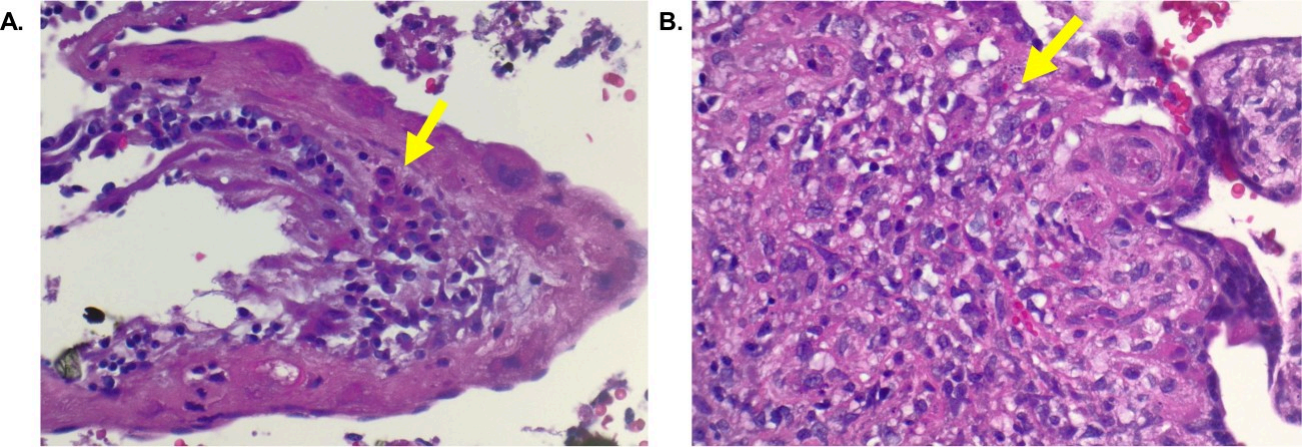
Histology of the placenta from a ZIKV-infected pregnant mother. Placental tissue from subject B1_0004 was stained with hematoxylin and eosin. Lymphocytes and macrophages are present in the chorionic villi (**A**-100X, **B**-400X). The arrow indicates inflammatory cells within a villus.

Lymphohistiocytic chronic villitis (inflammatory lesions in the placenta with an infiltrate of lymphocytes and macrophages) [80], was observed in the placentas of 5 of 11 (45%) ZIKV-infected mothers (Table 3 and Fig 2). The villitis was focal, involving less than 10 villi per focus, consistent with mild, low grade chronic villitis [73,74]. One placenta (B1_0004) demonstrated mild necrosis in the villitis focus and two placentas (B1_0004 and B1_0014) demonstrated small focal avascular villi with stromal fibrosis, consistent with fetal artery thrombosis in the absence of any other abnormality. We tested frozen placental samples by qRT-PCR but did not detect ZIKV RNA. In contrast, no vilitis was observed in any of the 8 ZIKV-uninfected subjects.

**Table 3:** Placental pathology. In 5 of 11 ZIKV-infected cases, focal villitis was observed as defined by less than 10 villi per focus.

Subject	Placental Histology Findings
**ZIKV infected**	
B1_0001	Villitis was not observed
B1_0002	Villitis was not observed
B1_0004	Villitis was observed in two foci, consistent with mild, low grade, chronic villitis of unknown etiology, occurrence of stromal fibrosis and occurrence of necrosis
B1_0005	Villitis was not observed
B1_0007	Villitis was not observed
B1_0008	Villitis was not observed
B1_0031	Villitis was not observed
B1_0027	Villitis was observed in two foci, consistent with mild, low grade, chronic villitis of unknown etiology
B1_0030	Villitis was observed in one focus, consistent with mild, low grade, chronic villitis of unknown etiology
B1_0014	Villitis was observed in two foci, consistent with mild, low grade, chronic villitis of unknown etiology and occurrence of stromal fibrosis
B1_0037	Villitis was observed in one focus, consistent with mild, low grade, chronic villitis of unknown etiology
**ZIKV uninfected**	
B1_0003	Villitis was not observed
B1_0009	Villitis was not observed
B1_0033	Villitis was not observed
B1_0026	Villitis was not observed
B1_0023	Villitis was not observed
B1_0016	Villitis was not observed
B1_0034	Villitis was not observed
B1_0015	Villitis was not observed

### Magnitude and kinetics of IgG binding responses to ZIKV and DENV over the course of pregnancy

**Fig 3:**
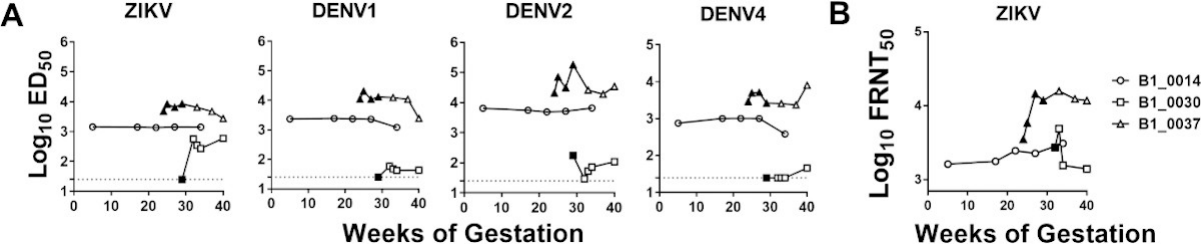
ZIKV binding and neutralizing IgG responses persist throughout pregnancy. **A.** Maternal plasma collected serially from three women diagnosed with ZIKV during pregnancy was assessed for IgG binding responses to ZIKV via virion capture ELISA. The estimated dilution at 50% of maximal binding (ED_50_) was calculated from serial dilutions of maternal plasma. Black filled points indicate time points when ZIKV viremia was detected by RT-PCR. **B.** ZIKV focus reduction neutralization titer throughout pregnancy.

ZIKV infection during pregnancy has been associated with prolonged viremia in humans and non-human primates [55,81–83], and one patient in our study (B1_0037) exhibited prolonged viremia, with plasma testing positive for ZIKV RNA up to 42 days post onset of symptoms (Fig 3). We compared ZIKV antibody binding dynamics between patient B1_0037 and two other ZIKV-infected women from the cohort for whom multiple sequential serum and urine samples were available for analysis (B1_0014 and B1_0030). B1_0030 only tested positive for ZIKV in urine by RT-PCR at the first 2 visits (within 18 days of symptoms), and B1_0014 tested ZIKV-negative by RT-PCR but was classified as ZIKV-infected by serology. Of note, these cases have different flavivirus exposure histories as B1_0014 had prior exposure to DENV, and B1_0030 had a primary ZIKV infection. The magnitude of maternal plasma IgG binding to ZIKV, DENV1, DENV2, and DENV4 was measured by virion capture ELISA and neutralization was measured by FRNT-50 in plasma collected at every visit during gestation and delivery (Fig 3). We found that all three subjects sustained high levels of flavivirus-binding IgG and neutralizing antibodies throughout pregnancy, with the peak antibody response detectable one to three weeks post onset of symptoms.

### Transplacental transfer of flavivirus-specific and cross-reactive IgG in ZIKV-infected and uninfected women

To determine if ZIKV infection during pregnancy disrupts transplacental transfer of flavivirus-specific IgG from mother to infant, we compared the magnitude of flavivirus-specific antibody binding responses in maternal plasma at delivery and infant cord blood plasma by virion capture ELISA in 20 mother-infant pairs with delivery samples available. For those with ZIKV infection, IgG binding to ZIKV, DENV1, DENV2, DENV3, and DENV4 virions was not significantly different between maternal plasma and paired infant cord blood from delivery (Wilcoxon Signed Rank Test; Bonferroni adjusted *P* >0.05 for all viruses tested).

**Fig 4:**
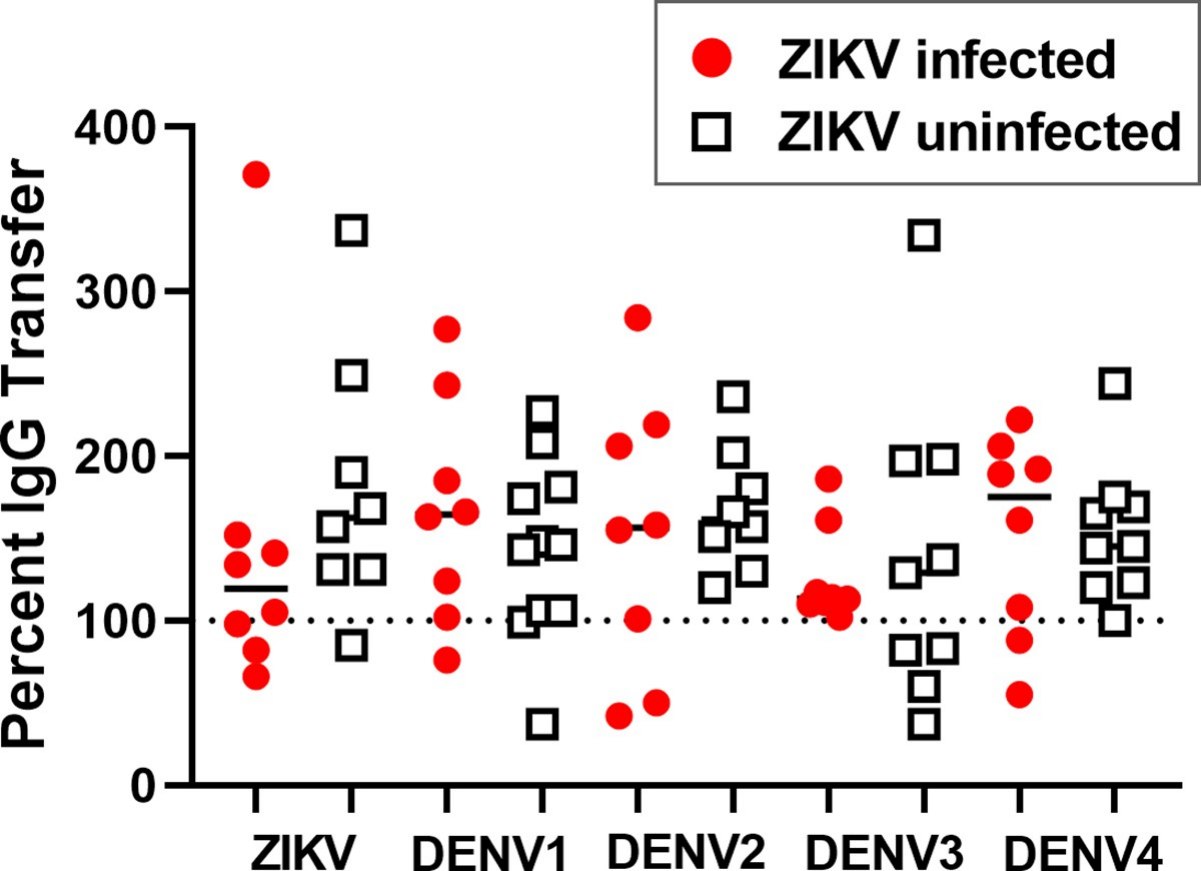
Efficient transplacental transfer of flavivirus-specific IgG. Plasma antibody binding to ZIKV, DENV1, DENV2, DENV3, and DENV4 was measured by virion capture ELISA using serial dilutions of maternal plasma and infant cord blood collected at delivery. The dilution at 50% of maximal binding (ED50) was calculated and the infant ED50 was assessed as a percentage of the maternal ED50 to yield percent transfer. Dotted line indicates 100% transfer and the solid line indicates the median. No significant differences in percent transfer were found in comparing ZIKV-infected and uninfected women for the all viruses tested by Exact Wilcoxon Rank Sum Test; Bonferroni adjusted *P* > 0.05 for all viruses tested.

We calculated the efficiency of mother-to-fetus transfer of flavivirus-specific IgG as the ratio of the magnitude of infant cord blood antibody binding response to the maternal response, expressed as a transfer efficiency percentage (Fig 4 and S3 Table). For those with paired maternal and infant samples available, we compared the flavivirus-specific IgG transfer efficiencies in ZIKV-infected (n = 8) and uninfected (n = 12) women, and found no significant difference in the transplacental transfer efficiency of flavivirus-specific IgG between the groups (Exact Wilcoxon Rank Sum Test; Bonferroni adjusted *P* > 0.05 for all viruses tested), indicating that ZIKV infection during pregnancy did not disrupt transplacental transfer of flavivirus-specific IgG.

As expected, in the virion capture ELISA we observed cross-reactive binding to ZIKV with plasma from 8 women who were DENV seropositive but ZIKV-uninfected. These ZIKV-uninfected subjects also demonstrated transfer of ZIKV-binding (cross-reactive, non-neutralizing) IgG from mother to infant (Fig 4 and S3 Table). As expected, we did not detect ZIKV-specific IgG in 2 ZIKV/DENV naïve subjects or in 2 DENV seropositive patients and therefore percent IgG transfer could not be calculated for these subjects. Of the 8 DENV seropositive subjects with ZIKV-reactive IgG transferred to cord blood, 5 were seropositive for multiple DENV serotypes (B1_0016, B1_0024, B1_0026, B1_0033, and B1_0034), and 2 were seropositive for only a single DENV serotype (B1_0009 and B1_0011), indicating that ZIKV cross-reactive IgG can be transferred to the fetus in the case of primary or secondary DENV exposure history. Moreover, percent IgG transfer was not significantly associated with magnitude of the type-specific IgG in maternal plasma (S2 Fig).

**Fig 5:**
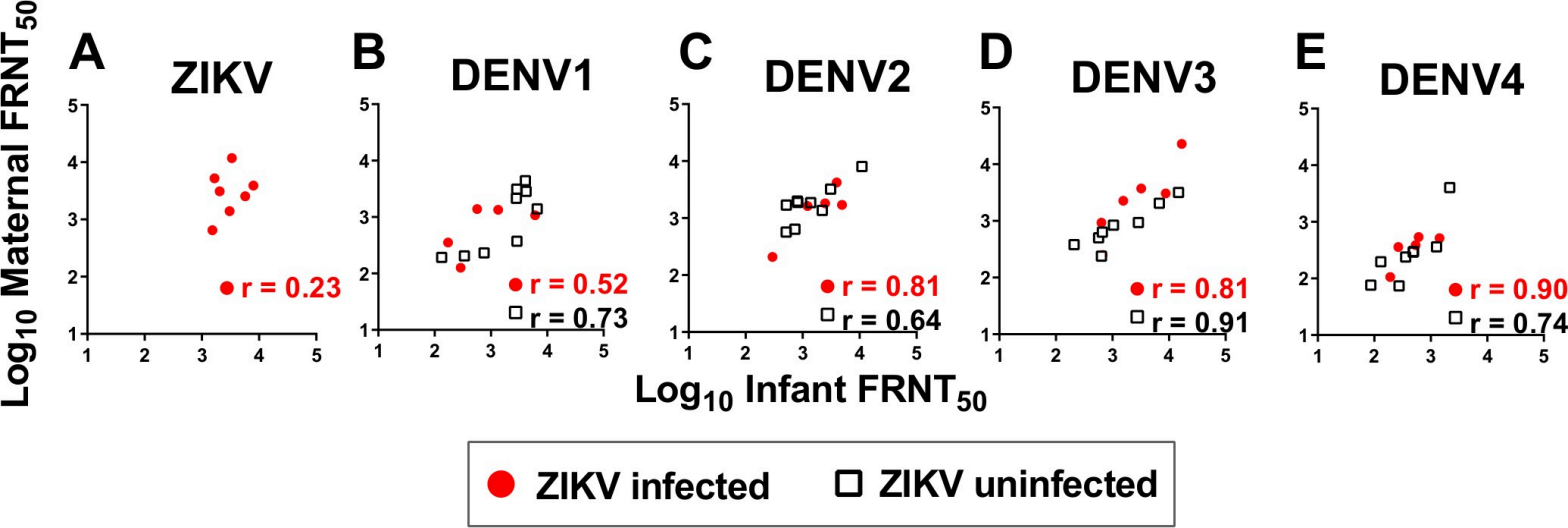
Maternal ZIKV infection does not disrupt transplacental transfer of DENV neutralizing IgG. Kendall Tau correlation of focus neutralization reduction titer-50 (FRNT-50) for maternal plasma and infant cord blood, separated by maternal ZIKV serostatus. Panels indicate correlation of maternal and infant neutralizing titers by flavivirus: ZIKV (A), DENV1 (B), DENV2 (C), DENV3 (D) and DENV4 (E). All correlations are *P*<0.05, except DENV1 and ZIKV in ZIKV-infected mothers where *P<*0.09 and *P<*0.45 respectively.

Additionally, we assessed whether there was efficient transplacental transfer of flavivirus neutralizing IgG in ZIKV-infected pregnant women. The DENV FRNT-50 of paired maternal and cord blood plasma also were positively correlated, suggesting that functional maternal IgG were transferred efficiently to the fetus (Fig 5).

### Transplacental transfer of vaccine-elicited IgG in ZIKV-infected and uninfected women

**Fig 6:**
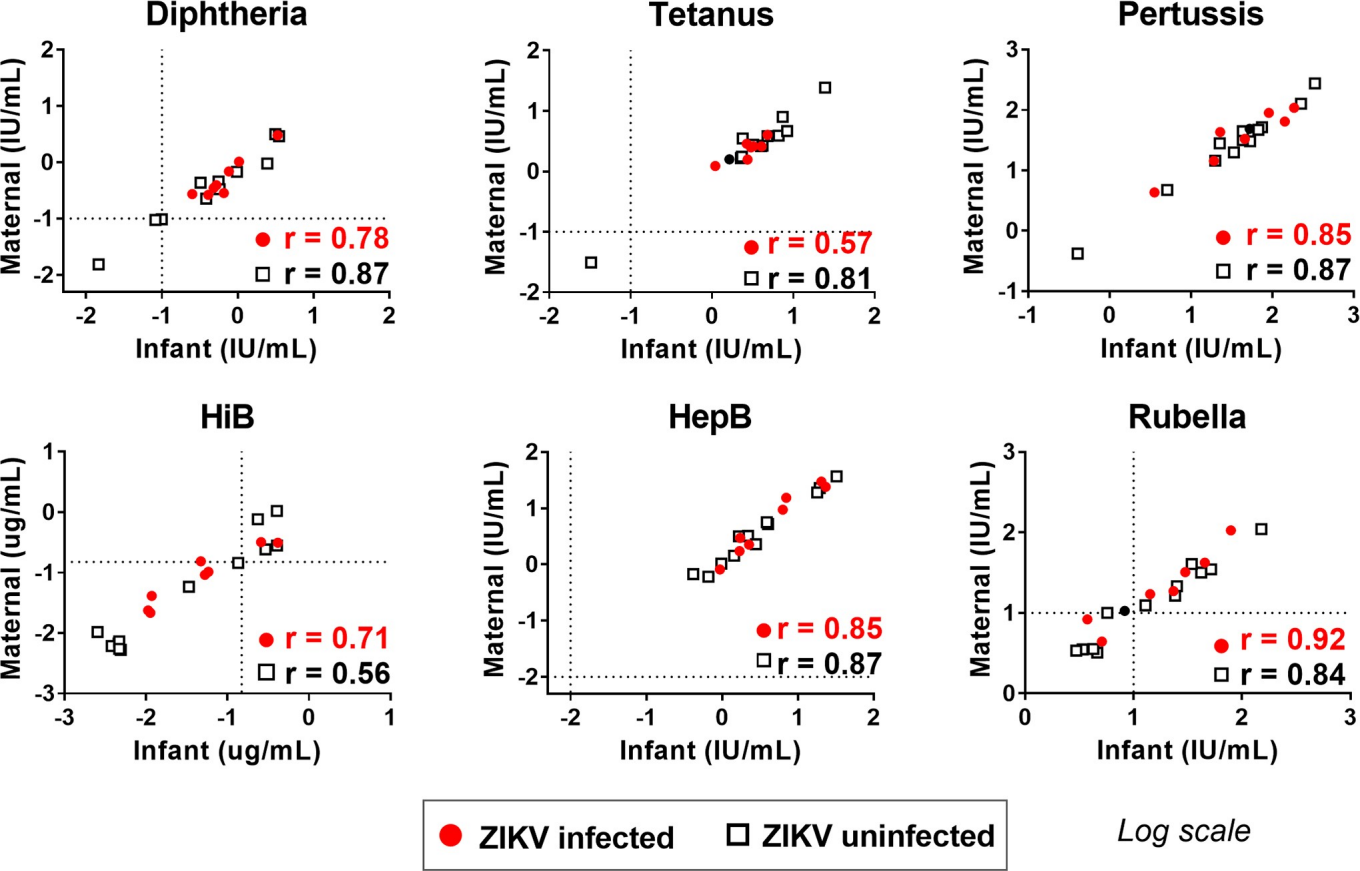
Strong correlation of maternal and infant vaccine-elicited IgG levels in ZIKV-infected mothers indicates efficient transplacental transfer during maternal ZIKV infection. IgG response to vaccine antigens in infant cord blood plasma and maternal plasma collected at delivery were measured by a binding antibody multiplex assay. Concentrations of vaccine-elicited IgG responses were calculated from reference sera standards as International Units (IU)/mL or μg/mL. ZIKV-infected (n = 8) and uninfected (n = 12) subjects are indicated in red and black respectively, and dotted lines denote WHO established protective IgG levels. Kendall Tau correlations were performed for each ZIKV infection group, with p<0.05 for all.

To assess whether ZIKV infection during pregnancy impacts placental transfer of IgG against vaccine antigens, we measured the magnitude of IgG binding against a panel of standard vaccine antigens from hepatitis B virus, rubella virus, *Haemophilus influenzae* type B, *Corynebacterium diphtheriae*, *Bordetella pertussis*, and *Clostridium tetani*. We found no significant differences in the magnitude of vaccine-specific IgG in maternal plasma and infant cord blood from delivery, in both ZIKV-infected and uninfected pregnant women (Wilcoxon Signed Rank Test, *P* > 0.05 for all vaccine antigens). Moreover, in ZIKV-infected and uninfected cases, we observed strong positive correlations in the concentration of vaccine-specific IgG between maternal plasma and infant cord blood for all vaccine antigens tested, indicating efficient placental transfer of vaccine-specific IgG levels regardless of ZIKV infection status (Fig 6 and S3 Table). Based on the protective vaccine-specific IgG levels established by the WHO, infants born to mothers who had protective levels of vaccine-specific IgG and ZIKV infection during pregnancy, received similarly protective IgG levels as infants born to ZIKV-naïve mothers [84].

Altogether our study demonstrates that efficient transfer of IgG from mother to fetus is maintained in this cohort irrespective of maternal ZIKV infection or placental pathology. Furthermore, this efficient transplacental IgG transfer includes both vaccine-specific antibodies and flavivirus antibodies relevant to maternal vaccination strategies and flavivirus disease in newborns.

## Discussion

Transplacental transfer of IgG provides passive immunity to fetuses, which is critical to protecting newborns in their first months of life [7]. However, maternal conditions and infections during pregnancy may disrupt IgG transfer via mechanisms including placental impairment and inflammatory responses [85]. Moreover, viral antigenic complexity and natural history of infection shapes the IgG populations elicited, which have different propensities to be transferred across the placenta by the FcRn [85,86]. Thus, we investigated the impact of maternal infection with ZIKV on maternal-fetal IgG transfer in 20 mother-infant pairs from a prospective cohort in Vitoria, Brazil. We assessed transfer of key IgG populations, including ZIKV and DENV binding and neutralizing IgG, as well as IgG specific to routine vaccine antigens.

For all flavivirus and vaccine antigens tested, we found that maternal and infant binding IgG levels were highly correlated in both ZIKV-infected and -uninfected groups. Also, there were no significant differences in the magnitude of flavivirus-binding IgG levels between mothers and infants among mothers with ZIKV infection during pregnancy. Moreover, DENV neutralization and binding IgG levels were highly correlated between mothers and infants regardless of maternal ZIKV infection in pregnancy. In the case of DENV1 and ZIKV neutralizing IgG levels, though the positive correlation between mother and infant neutralizing titer was weak (r = 0.23 and 0.52 respectively), the outliers of the linear trend were shifted such that the magnitude of infant IgG neutralization is greater than that of the maternal neutralizing titer, indicating efficient IgG transfer. This positive association of maternal and infant IgG levels represents active transfer that is not solely dependent on the magnitude of the type-specific IgG in maternal plasma. Also, the substantially overlapping ranges in antibody levels between mothers and infants suggests no biologically relevant differences in transplacental transfer of flavivirus binding and neutralizing IgG, or of vaccine specific IgG after ZIKV infection in pregnancy. Cumulatively, these data indicate no evidence of impairment in the transplacental IgG transfer at the time of birth after maternal ZIKV infection during pregnancy, as compared to mothers with fever and rash during pregnancy without ZIKV infection.

Our study corroborates recent findings demonstrating efficient transfer of ZIKV, DENV3 and DENV4 neutralizing antibodies in mother-infant pairs from the Northeast of Brazil in 2016 [61]. Specifically, Castanha et al. found that newborns with the outcome of micrcephaly, some of whom were exposed to ZIKV in utero, had no evidence of impaired transfer of neutralizing antibodies at birth as compared to controls without microcephaly [61]. Our work complements the finding from that case-control study through a prospective cohort design, in which we identified women with ZIKV infection during pregnancy and followed up until delivery to quantify impact on transplacental IgG transfer. This prospective design adds a temporality to the association between ZIKV infection and neutralizing IgG transfer oberserved earlier [61]. Moreover, our study represents a geographically distinct site in Southeast Brazil, with lower ZIKV prevalence. Altogether, this work strengthens the body of evidence indicating no impairment in transplacental IgG transfer with ZIKV infection in pregnancy, with implications for maternal vaccination strategies and flavivirus disease in newborns.

As different viral antigen-specific IgG subpopulations may be differentially impaired in placental transfer due to maternal infections and conditions during pregnancy [87], we tested IgG transfer of non-flavivirus antibodies that are specific to diverse vaccine antigens. IgG elicited by routine pediatric and boosted maternal vaccines were also transferred efficiently despite maternal ZIKV infection. In cases where the mother had a protective level of IgG against vaccine-preventable infections, the infant received a similarly protective level.

Our study further aimed to complement existing evidence of placental pathology caused by ZIKV infection, and determine whether this could have a role in the transplacental transfer of humoral immunity. Previous observations of impaired transplacental IgG transfer in the setting of maternal HIV and malaria infection generally have been noted in conjunction with identifiable placental pathology [40,85]. Although we were unable to detect ZIKV RNA in placentas from our study, another study identified ZIKV RNA in 54% of placentas from 44 ZIKV-infected women [48]. We found that 5 of 11 ZIKV-infected women in our cohort had chronic placental villitis, higher than the 5–15% expected for term placentas [88]. Notably, this pathology is similar to that described in placental infection with HCMV, rubella virus, or *Toxoplasma gondii* [80]. In constrast, no vilitis was observed in the 8 placentas assessed from ZIKV-uninfected mothers, suggesting that vilitis in the ZIKV-infected subjects may have been specific to maternal ZIKV infection in pregnancy. Furthermore, to assess the impact of ZIKV infection associated placental pathology on IgG transfer, subgroups of ZIKV-infected subjects with noted placental pathology would have to be compared to a ZIKV-infected subgroup without placental pathology. However, our limited sample size of 8 ZIKV-infected individuals with paired infant samples precludes formal comparison

We found that despite disruption of placental architecture in nearly half of our ZIKV-infected pregnancies, transplacental transfer of flavivirus-binding and -neutralizing IgG was sustained following maternal ZIKV infection. This finding is relevant to future studies of vaccine-elicited fetal protection against ZIKV, as animal studies demonstrate envelope binding and neutralizing antibodies as correlates of protection against ZIKV infection [89,90]. Transfer of flavivirus-neutralizing antibody is relevant because neutralization titers are known to correlate with vaccine protection against other flaviviruses, including Japanese encephalitis virus, yellow fever virus, West Nile virus, and tick-borne encephalitis virus [91–94]. Moreover, in ZIKV-infected women with serial plasma collection during pregnancy, ZIKV-specific IgG levels were sustained throughout gestation after peak response within 3 weeks of symptoms. These kinetics suggest that transfer of flavivirus-specific IgG to the fetus should readily occur throughout the 2^nd^ and 3^rd^ trimesters of pregnancy following maternal ZIKV infection. While it is possible that ZIKV infection during pregnancy could result in a transient disruption of transplacental IgG transfer that is restored by the time of birth, our goal was to evaluate levels of maternal IgG present at delivery as these transferred IgG have the potential to modulate protection or disease risk in infants [7–10,18,34,95].

There are several implications of the findings in this study. Efficient transfer of ZIKV-neutralizing IgG in ZIKV-infected mothers could be a mode of transferring protective humoral immunity from mother to infant, despite infection during pregnancy. Notably, transfer of protective levels of vaccine-specific IgG to boost passive immunity in the newborn is a key objective of maternal immunization [96] and our findings suggest that ZIKV infection during pregnancy does not impair this protective mechanism. With candidate maternal ZIKV vaccines or therapeutics, this may be one mode of conferring passive immunity to the fetus and, potentially, reducing the burden of congenital and neonatal ZIKV infection.

Alternatively, transfer of cross-reactive non-neutralizing DENV-elicited antibodies may pose a risk as antibodies from primary DENV infection can enhance secondary DENV infection, leading to more severe disease in infants as maternal antibody titers wane [20,25]. We detected transplacental transfer of ZIKV-binding IgG in DENV-immune mothers without ZIKV infection. Cross-reactive ZIKV-elicited antibodies may be able to mediate antibody-dependent enhancement of subsequent DENV infection in early infancy [20,27,97–99]. Additionally, there is some concern that efficient IgG transfer may facilitate transcytosis of ZIKV-IgG complexes into the fetal compartment, a suggested mechanism of fetal infection for HCMV [18,32]. Thus, transplacental transfer of ZIKV cross-reactive IgG should be considered in the evaluation of candidate ZIKV vaccines, as sub-neutralizing levels of cross-reactive IgG may increase the risk of severe flavivirus infections in fetuses or infants.

Limitations of this study include the small sample size of 26 mothers, including 20 mother-infant pairs with delivery samples available. In assessing statistically significant associations (alpha = 0.05) via Kendall-Tau correlations between mothers and infants, we had 89% to 99% power across assays to detect a true direct correlation of maternal and infant IgG levels. Therefore, our conclusions of intact placental IgG transfer are predominantly based on the high levels of association of maternal and cord blood antibody responses.

As for significant differences in the magnitude of IgG responses between ZIKV-infected and uninfected mother-infants pairs via the Wilcoxon Signed-Rank Test, this study has a 93% power to detect differences in flavivirus binding responses between ZIKV-infected and uninfected groups (alpha = 0.05). However, the study is underpowered (power < 50%) to detect significant differences in magnitude of neutralizing or vaccine-specific responses between ZIKV-infected and -uninfected subjects due to higher levels of variability in these measures. Consequently, significant differences are only reported and analyzed for the flavivirus-binding IgG levels, but not for neutralization and vaccine-elicited IgG levels. Though, noting the substantially overlapping range of immune responses in the ZIKV-infected group as compared to the ZIKV-uninfected group is biologically relevant to our understanding of transplacental IgG transfer in the setting of maternal ZIKV infection and could inform future studies on neonatal flavivirus immunity.

Another limitation of our study is the challenge of determining whether subjects were truly exposed to ZIKV during pregnancy, as symptoms could have resulted from other infections and/or ZIKV infection could have occurred prior to pregnancy. Since viremia may have subsided by the time of study enrollment, we developed an algorithm to define ZIKV infection serologically, even in the context of cross-reactive antibodies from prior DENV infection. This algorithm and ZIKV case definition were based on the rational assumption that ZIKV seropositivity resulted from a recent infection (i.e. during pregnancy) due to the timing of our study relative to the introduction of ZIKV into Brazil. This assumption will not apply in future studies, since the high force of infection during the 2015–2017 outbreak and the potential for subsequent endemic transmission mean many women will already be ZIKV seropositive before pregnancy. Moreover, this study reflects the findings in a symptomatic pregnancy cohort, whereas the majority of ZIKV infections are asymptomatic[100–102].

In summary, this study demonstrates efficient transplacental transfer of IgG specific to diverse flavivirus and routine vaccine antigens following ZIKV infection during pregnancy in a unique prospective mother-infant cohort from the Latin American ZIKV outbreak. Transplacental transfer of ZIKV-specific IgG in pregnancy may contribute to protection of the fetus from congenital Zika syndrome and the infant from ZIKV infection. However, efficiently transferred IgG might mediate adverse effects in infants including increased risk of severe DENV in infancy, as well as potentially mediating FcRn-dependent transfer of ZIKV immune complexes into the fetal compartment. The relationship between efficient maternal IgG transfer and reduced or enhanced congenital infection or disease remains to be further elucidated. Delineating ZIKV-specific IgG levels and function that favor fetal and neonatal protection will be key for guiding a strategic timeline for pediatric vaccine boosts, timing of vaccine administration during pregnancy, and dosing of antibody therapies targeted for pregnancy. Longitudinal investigations of neonatal immunity, in the context of transplacental transfer of flavivirus antibodies will be a valuable area of investigation to define serological mediators of risk or protection for infants. Given the uncertain benefits or risks of efficient transfer of flavivirus IgG, ZIKV and DENV vaccine strategies will need to carefully consider the timing and type of vaccination and boosting in order to maintain protective levels of antibodies in women of reproductive age and infants.

## Supporting information

S1 Fig Study design flow chart indicating that 26 symptomatic pregnant women were eligible and enrolled into prospective cohort, and only 20 mother-infant pairs were analyzed due to paired sample availability. The 20 pairs were classified into two groups based on ZIKV exposure status during pregnancy. Accordingly, 8 mothers were determined to be infected with ZIKV during pregnancy and 12 were not. Laboratory tests were conducted on all available mother and infant samples without further stratification.
(TIF)

S2 Fig Lack of associations between magnitude of maternal flavivirus-specific IgG and percent IgG transfer. A post-hoc Spearman correlation analysis was conducted to assess whether higher magnitude of maternal flavivirus IgG due to recency of infection during pregnancy could be driving efficient transplacental IgG transfer. No strong positive associations were noted, suggesting that magnitude of maternal IgG alone does not predict percent transfer for each antigen shown (Unadjusted p-values > 0.05 for ZIKV, DENV1, DENV2, DENV4 and <0.038 for DENV3).
(TIF)

S1 Table Clinical results of prenatal screening for TORCH infections. These data from maternal serum samples from pregnany indicate no recent *Toxoplasma* infections and high seropositivity to rubella virus as tested by chemiluminescent microparticle immunoassays, Also, there was no evidence for syphillis, which was assessed by the VDRL test. Infant cordblood qPCR testing for CMV indicates one potential case of congential CMV transmission in the ZIKV-uninfected group. Proportion of mothers or infants with postive test results are reported as the numerator, whereas the denominator is the number of the total samples tested.
(DOCX)

S2 Table Timeline of infection for each enrolled mother in this study. (DOCX)

S3 Table Transplacental transfer efficiency. Median and range shown for mothers and infants by each flavivirus binding, vaccine antigen binding or flavivirus neutralizing response measured. Subjects are grouped by maternal ZIKV infection status to facilitate comparison of the magnitudes of antibody responses and calculated IgG percent transfer. Infant antibody response as a portion of maternal response are indicated in the percent transfer column, where median, range and number of mother-infant pairs per group are shown. Bonferroni adjusted p-values shown from Wilcoxon Signed Rank Tests to assess significant differences in the mother to infant percent transfer of antibodies in the ZIKV-infected versus uninfected groups. No significant differences in the percent transfer of flavivirus binding antibody responses between mother and infant were observed regardless of ZIKV serostatus. NP indicates that a p-value is not shown since this study is not powered to detect significant differences between mothers and infants for those antigens.
(DOCX)
