# Supplementary figures and images for: Efficient transplacental IgG transfer in women infected with Zika virus during pregnancy
Source: PLoS Negl Trop Dis. 2019 Aug 26;13(8):e0007648. doi: 10.1371/journal.pntd.0007648 (PMC6730934; doi:10.1371/journal.pntd.0007648)

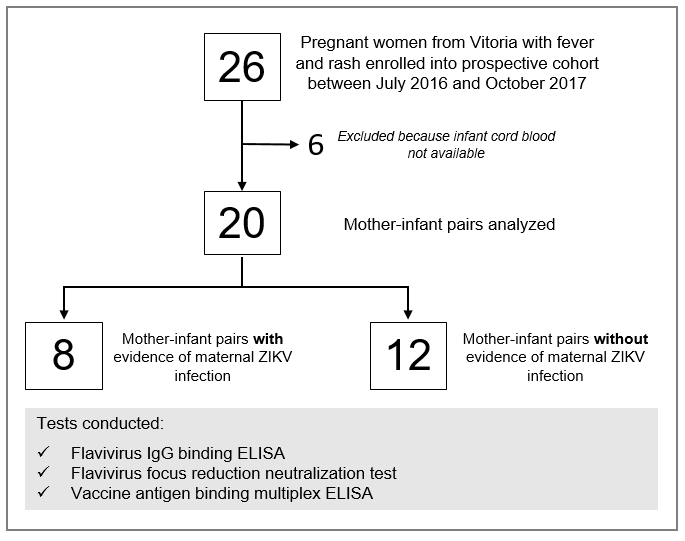

Supplement: S1 Fig — The 20 pairs were classified into two groups based on ZIKV exposure status during pregnancy. Accordingly, 8 mothers were determined to be infected with ZIKV during pregnancy and 12 were not. Laboratory tests were conducted on all available mother and infant samples without further stratification. (TIF) [file pntd.0007648.s001.tif]

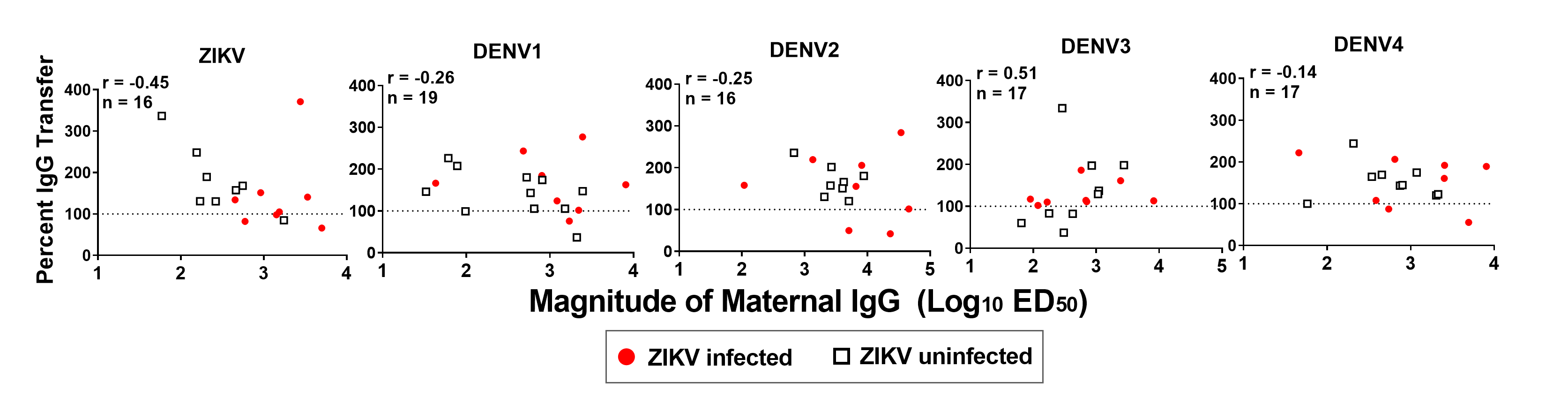

Supplement: S2 Fig — A post-hoc Spearman correlation analysis was conducted to assess whether higher magnitude of maternal flavivirus IgG due to recency of infection during pregnancy could be driving efficient transplacental IgG transfer. No strong positive associations were noted, suggesting that magnitude of maternal IgG alone does not predict percent transfer for each antigen shown (Unadjusted p-values > 0.05 for ZIKV, DENV1, DENV2, DENV4 and <0.038 for DENV3). (TIF) [file pntd.0007648.s002.tif]
