# Supplementary material for: Efficient transplacental IgG transfer in women infected with Zika virus during pregnancy
Source: PLoS Negl Trop Dis. 2019 Aug 26;13(8):e0007648. doi: 10.1371/journal.pntd.0007648 (PMC6730934; doi:10.1371/journal.pntd.0007648)
Supplement: S1 Table — These data from maternal serum samples from pregnany indicate no recent Toxoplasma infections and high seropositivity to rubella virus as tested by chemiluminescent microparticle immunoassays, Also, there was no evidence for syphillis, which was assessed by the VDRL test. Infant cordblood qPCR testing for CMV indicates one potential case of congential CMV transmission in the ZIKV-uninfected group. Proportion of mothers or infants with postive test results are reported as the numerator, whereas the denominator is the number of the total samples tested. (DOCX) [file pntd.0007648.s003.docx]

|  | **ZIKV-infected** | **ZIKV-uninfected** | **Unavailable** |
| --- | --- | --- | --- |
| **Toxoplasma IgM** | 0/8 | 0/9 | 3/20 |
| **Toxoplasma IgG** | 3/8 | 7/10 | 2/20 |
| **Rubella IgG** | 6/7 | 9/9 | 4/20 |
| **Cord blood**  **CMV viremia** | 0/7 | 1/11 | 2/20 |
| **Syphilis VDRL** | 0/8 | 0/10 | 2/26 |

# S1 Table: Clinical results of prenatal screening for TORCH infections. These data from maternal serum samples from pregnany indicate no recent Toxoplasma infections and high seropositivity to Rubella as tested by chemiluminescent microparticle immunoassays, Also, there was no evidence for Syphillis, which was assessed by the VDRL test. Infant cordblood qPCR testing of CMV indicates one potential case of congential CMV transmission in the ZIKV-uninfected group. Proportion of patient mothers or infants with postive test results are reported as the numerator, whereas the denominator is the number of the total samples tested.
