# Supplementary material for: Efficient transplacental IgG transfer in women infected with Zika virus during pregnancy
Source: PLoS Negl Trop Dis. 2019 Aug 26;13(8):e0007648. doi: 10.1371/journal.pntd.0007648 (PMC6730934; doi:10.1371/journal.pntd.0007648)
Supplement: S2 Table — (DOCX) [file pntd.0007648.s004.docx]

# S2 Table. Timeline of infection for each enrolled mother in this study.

| **ID** | **Age** | **Gestational Age (days) at symptom onset** | **Trimester of Infection** | **Days between first sample collection and symptom onset** | **ZIKV serum RT-PCR Results** | **DENV serum RT-PCR Results** |
| --- | --- | --- | --- | --- | --- | --- |
| B1_0001 | 38 | 73 | 1 | 4 | + | N/A |
| B1_0002 | 29 | 97 | 2 | 4 | + | N/A |
| B1_0003 | 28 | 66 | 1 | 7 | - | N/A |
| B1_0004 | 21 | 94 | 2 | 4 | + | N/A |
| B1_0005 | 26 | 31 | 1 | 3 | + | N/A |
| B1_0006 | 22 | 68 | 1 | N/A | N/A | N/A |
| B1_0007 | 29 | 78 | 1 | N/A | N/A | N/A |
| B1_0008 | 22 | 95 | 2 | 2 | + | N/A |
| B1_0009 | 31 | 40 | 1 | 4 | + | N/A |
| B1_0011 | 26 | 232 | 3 | N/A | - | - |
| B1_0014 | 19 | 27 | 1 | 4 | - | - |
| B1_0015 | 34 | 117 | 2 | 8 | - | N/A |
| B1_0016 | 35 | 99 | 2 | N/A | N/A | N/A |
| B1_0019 | 28 | 157 | 2 | 15 | - | N/A |
| B1_0021 | 30 | 75 | 1 | 3 | - | N/A |
| B1_0023 | 18 | 167 | 2 | 7 | - | N/A |
| B1_0024 | 30 | 136 | 2 | N/A | N/A | N/A |
| B1_0026 | 31 | 224 | 3 | N/A | N/A | N/A |
| B1_0027 | 23 | 179 | 2 | 14 | - | - |
| B1_0030 | 20 | 203 | 3 | 2 | + | N/A |
| B1_0031 | 39 | 257 | 3 | 4 | + | N/A |
| B1_0033 | 19 | 174 | 2 | 9 | - | - |
| B1_0034 | 28 | 173 | 2 | 2 | - | N/A |
| B1_0035 | 32 | 176 | 2 | 3 | - | - |
| B1_0037 | 19 | 159 | 2 | 4 | + | N/A |
| B1_0039 | 29 | 225 | 3 | N/A | N/A | N/A |
