# Supplementary material for: Efficient transplacental IgG transfer in women infected with Zika virus during pregnancy
Source: PLoS Negl Trop Dis. 2019 Aug 26;13(8):e0007648. doi: 10.1371/journal.pntd.0007648 (PMC6730934; doi:10.1371/journal.pntd.0007648)
Supplement: S3 Table — Median and range shown for mothers and infants by each flavivirus binding, vaccine antigen binding or flavivirus neutralizing response measured. Subjects are grouped by maternal ZIKV infection status to facilitate comparison of the magnitudes of antibody responses and calculated IgG percent transfer. Infant antibody response as a portion of maternal response are indicated in the percent transfer column, where median, range and number of mother-infant pairs per group are shown. Bonferroni adjusted p-values shown from Wilcoxon Signed Rank Tests to assess significant differences in the mother to infant percent transfer of antibodies in the ZIKV-infected versus uninfected groups. No significant differences in the percent transfer of flavivirus binding antibody responses between mother and infant were observed regardless of ZIKV serostatus. NP indicates that a p-value is not shown since this study is not powered to detect significant differences between mothers and infants for those antigens. (DOCX) [file pntd.0007648.s005.docx]

S3 Table: Transplacental transfer efficiency. Median and range shown for mothers and infants by each flavivirus binding, vaccine antigen binding or flavivirus neutralizing response measured. Subjects are grouped by maternal ZIKV infection status to facilitate comparison of the magnitudes of antibody responses and calculated IgG percent transfer. Infant antibody response as a portion of maternal response are indicated in the percent transfer column, where median, range and number of mother-infant pairs per group are shown. Bonferroni adjusted p-values shown from Wilcoxon Signed Rank Tests to assess significant differences in the mother to infant percent transfer of antibodies in the ZIKV-infected versus uninfected groups. No significant differences in the percent transfer of flavivirus binding antibody responses between mother and infant were observed regardless of ZIKV serostatus. NP indicates that a p-value is not shown since this study is not powered to detect significant differences between mothers and infants for those antigens.

|  | **ZIKV infected** | | | **ZIKV uninfected** | | |  |
| --- | --- | --- | --- | --- | --- | --- | --- |
| **Antigen** | **Mother** | **Infant** | **Percent Transfer** | **Mother** | **Infant** | **Percent Transfer** | **Adjusted P-Value for Percent Transfer** |
| **Virion Binding (ED50)** | | | | | | | |
| ZIKV | 1539.27, (450.7, 5008.16) | 1507.09, (489.46, 10233.26) | 119.54, (66.07, 371.19), 8 | 154.64, (25, 2041.77) | 283.81, (25, 1484.14) | 162.81, (84.82, 336.79), 8 | 0.762 |
| DENV1 | 1708.6, (43, 8077.16) | 1495.16, (71.54, 13149.29) | 164.58, (75.9, 277.32), 8 | 392.22, (25, 2469.76) | 730.09, (25, 3645.73) | 146.22, (36.75, 226.63), 11 | 0.983 |
| DENV2 | 8148.65, (109.44, 45907.79) | 10003.6, (173.26, 97705.33) | 156.88, (42, 283.99), 8 | 1400.42, (25, 8790.74) | 3383.9, (25, 15865.84) | 161.65, (120, 235.81), 8 | 0.983 |
| DENV3 | 578.3, (25, 8226) | 783.45, (104.8, 9255) | 113.18, (101.68, 185.72), 8 | 288.5, (25, 2758) | 249.45, (25, 5474) | 129.2, (36.67, 334), 9 | 1 |
| DENV4 | 648.46, (45.79, 8052.2) | 2039.78, (101.75, 15258.81) | 175.2, (55.42, 222.18), 8 | 207.29, (25, 2134.25) | 666.41, (25, 2623.53) | 144.69, (100.19, 244.36), 9 | 1 |
| **Vaccine Antigen Binding** | | | | | | | |
| HepB (IU/mL) | 6.21, (0.81, 29.81) | 4.29, (0.93, 23.26) | 82.75, (45.49, 114.8), 8 | 3.19, (0.6, 36.84) | 2.5, (0.42, 32.37) | 86.59, (52.66, 122.69), 12 | NP |
| HiB (ug/mL) | 0.1, (0.02, 0.32) | 0.05, (0.01, 0.42) | 54.46, (28.64, 133.18), 8 | 0.03, (0.01, 1.05) | 0.02, (0, 0.41) | 66.52, (24.43, 145.18), 12 | NP |
| Pertussis (IU/mL) | 46.05, (4.32, 108.94) | 49.25, (3.57, 184.88) | 120.4, (52.67, 221.16), 8 | 30.97, (0.42, 275.69) | 43.27, (0.41, 333.33) | 137.31, (80.7, 178.04), 12 | NP |
| Tetanus (IU/mL) | 2.55, (1.23, 4.03) | 2.88, (1.1, 4.82) | 120.27, (89.03, 174.24), 8 | 3.13, (0.03, 24.39) | 4.1, (0.03, 24.58) | 129.52, (68.4, 180.58), 12 | NP |
| Diphtheria (IU/mL) | 0.37, (0.26, 3.03) | 0.59, (0.25, 3.35) | 121.79, (91.6, 230.74), 8 | 0.38, (0.02, 3.16) | 0.47, (0.01, 3.5) | 112.48, (74.29, 257.27), 12 | NP |
| Rubella (IU/mL) | 17.92, (4.43, 106.41) | 18.8, (3.74, 78.79) | 87.89, (44.9, 125.33), 8 | 14.39, (3.21, 110.04) | 18.54, (2.96, 151.11) | 118.36, (57.08, 148.89), 12 | NP |
| **Neutralizing Titer (FRNT-50)** | | | | | | | |
| DENV1 | 1348, (25, 2723) | 567, (25, 6086) | 115.62, (41.12, 564.04), 6 | 205, (25, 4417) | 1820.5, (25, 6631) | 156.37, (68.91, 775.13), 8 | NP |
| DENV2 | 1711, (25, 8019) | 2478, (25, 10957) | 136.47, (75.32, 287.61), 6 | 640, (25, 3222) | 770.5, (25, 3111) | 82.2, (30.6, 162.78), 8 | NP |
| DENV3 | 2270, (25, 22873) | 1554, (25, 16742) | 79.68, (68.46, 288.15), 6 | 380, (25, 4735) | 652.5, (25, 14689) | 196.38, (55.26, 463.08), 8 | NP |
| DENV4 | 491, (25, 4029) | 537, (25, 2159) | 125.81, (53.59, 278.95), 6 | 74, (25, 362) | 286, (25, 1260) | 165.89, (65.99, 371.62), 7 | NP |
